# Exposure to hypoxia causes stress erythropoiesis and downregulates immune response genes in spleen of mice

**DOI:** 10.1186/s12864-021-07731-x

**Published:** 2021-06-05

**Authors:** Haijing Wang, Daoxin Liu, Pengfei Song, Feng Jiang, Xiangwen Chi, Tongzuo Zhang

**Affiliations:** 1 Key Laboratory of Adaptation and Evolution of Plateau Biota, Northwest Institute of Plateau Biology, Chinese Academy of Sciences, Xining, 810001 Qinghai China; 2 Medical College of Qinghai University, Xining, 810016 Qinghai China; 3 Qinghai Provincial Key Laboratory of Animal Ecological Genomics, Xining, 810008 Qinghai China; 4 University of Chinese Academy of Sciences, Beijing, 100049 China

**Keywords:** Transcriptome, Spleen, Stress erythropoiesis, Immune response, Hypoxia

## Abstract

**Background:**

The spleen is the largest secondary lymphoid organ and the main site where stress erythropoiesis occurs. It is known that hypoxia triggers the expansion of erythroid progenitors; however, its effects on splenic gene expression are still unclear. Here, we examined splenic global gene expression patterns by time-series RNA-seq after exposing mice to hypoxia for 0, 1, 3, 5, 7 and 13 days.

**Results:**

Morphological analysis showed that on the 3rd day there was a significant increase in the spleen index and in the proliferation of erythroid progenitors. RNA-sequencing analysis revealed that the overall expression of genes decreased with increased hypoxic exposure. Compared with the control group, 1380, 3430, 4396, 3026, and 1636 genes were differentially expressed on days 1, 3, 5, 7 and 13, respectively. Clustering analysis of the intersection of differentially expressed genes pointed to 739 genes, 628 of which were upregulated, and GO analysis revealed a significant enrichment for cell proliferation. Enriched GO terms of downregulated genes were associated with immune cell activation. Expression of *Gata1*, *Tal1* and *Klf1* was significantly altered during stress erythropoiesis. Furthermore, expression of genes involved in the immune response was inhibited, and NK cells decreased.

**Conclusions:**

The spleen of mice conquer hypoxia exposure in two ways. Stress erythropoiesis regulated by three transcription factors and genes in immune response were downregulated. These findings expand our knowledge of splenic transcriptional changes during hypoxia.

**Supplementary Information:**

The online version contains supplementary material available at 10.1186/s12864-021-07731-x.

## Background

The spleen contains two compartments: the white pulp (WP) and the red pulp (RP). The WP embeds with multiple lymph node-like structures and is involved in the defense against blood-borne pathogens [1]. Adaptive and innate immune cells localize in specific areas in the spleen to orchestrate the immune response [2]. The RP removes aged, dead or opsonized cells from the circulation. The spleen is also a reservoir of red blood cells (RBC), and can store 15–25% of the total RBC volume [3, 4]. Hematopoietic stem cells (HSCs) are also found in the RP of the murine spleen [5]. Physiological or clinical conditions that reduce tissue oxygen tension can trigger stress erythropoiesis in the spleen [6], and the spleen servers as a niche for HSCs [7].

The spleen is associated with adaptation to hypoxia and hypoxic stress. In response to exercise, apnea, or simulated altitude, stored RBCs are ejected and the volume of the spleen decreases in humans [8, 9]. Individuals living at high altitude, like Sherpas and mountain climbers who have summited mount Everest, have larger spleen volumes [10, 11]. Animal studies have shown that up to 40% of the increased RBCs may originate in a tonic contraction of the spleen during hypoxia [12]. The spleen weight, cell counts and components of the WP and RP also changed during hypoxia [13–17]. Additional experiments are required for further understanding molecular mechanisms underlying stress erythropoiesis, which is often referred to as splenic erythropoiesis in mice model [18].

Erythropoietin (EPO)- and Bone morphogenetic protein 4 (BMP4)-dependent pathways regulate erythrocyte differentiation. EPO is the main regulator of red cell production in both the basal and stress state. After 12 h of exposure to severe hypoxia, EPO serum levels increase 300% with respect to the control value [19]. Expression of BMP4, induced by EPO [20] and primarily regulated by *Hif2α*, has been identified as a key signal involved in stress erythropoiesis, especially in phenylhydrazine (PHZ)-induced acute anemia [21–24]. Interestingly, the hypoxic and immune responses are interconnected [25], and pro-inflammatory cytokines can trigger erythropoiesis [26–28]. However, our understanding of the mechanisms underlying stress erythropoiesis, and of splenic immune responses during hypoxia, is incomplete. Moreover, global changes in gene expression have not been sufficiently investigated. In this study, we used time-series RNA-seq to investigate transcriptional changes in the murine spleen at different time points during hypoxic treatment and findings of the present study provide evidence that *Gata1*, *Tal1* and *Klf1* promote stress erythropoiesis and immune response genes downregulated.

## Results

### Hypoxia induces splenomegaly and splenic erythropoiesis

**Fig. 1:**
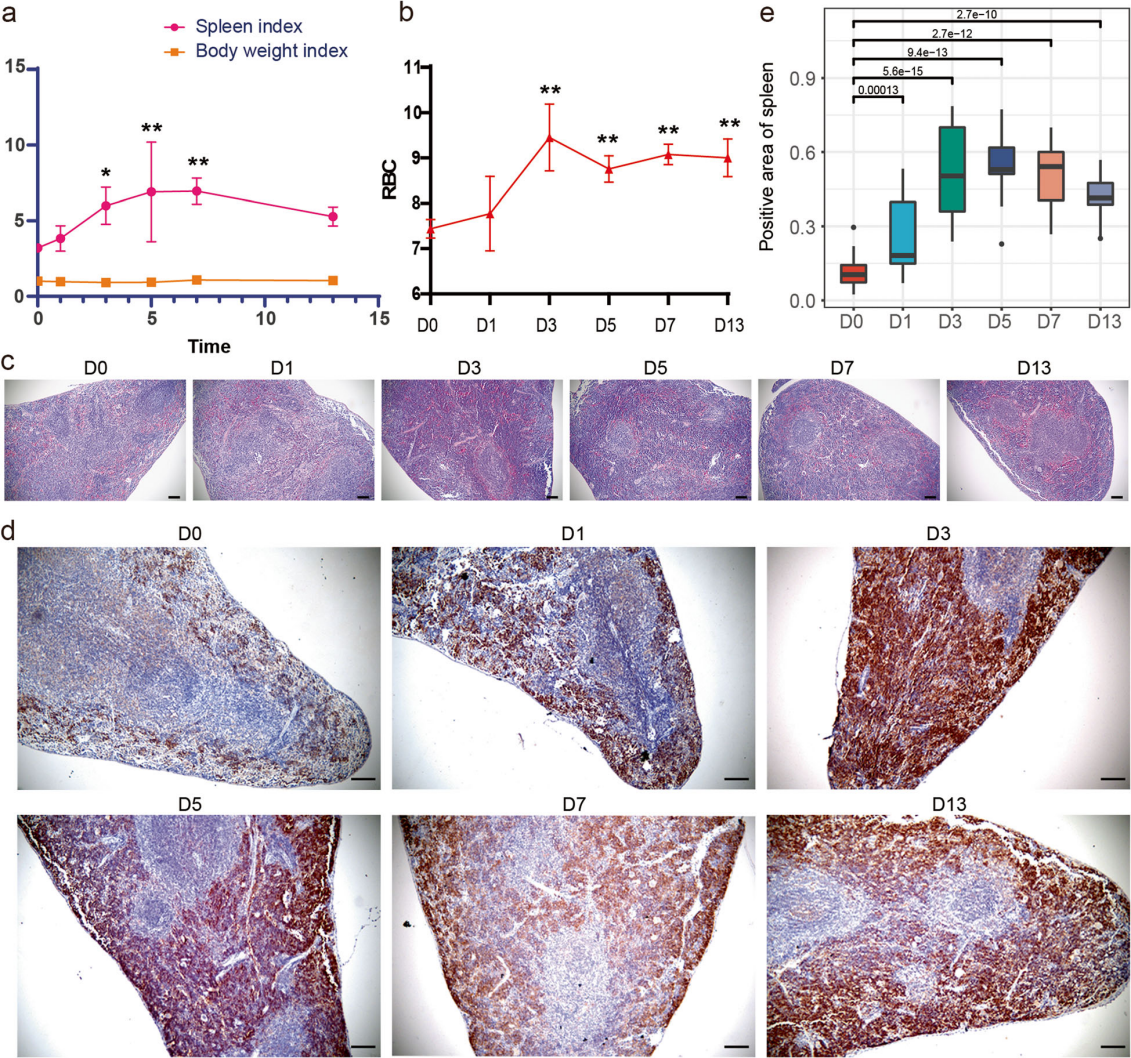
Histological analyses of the spleen. **a** Spleen index were calculated by one-way ANOVA (*P* = 0.02) and followed by LSD multiple comparison test (*p* = 0.033, 0.007, 0.006, respectively). Spleen index and the body weight index were calculated as described in Methods. **b** RBC counts of mice were calculated on the normoxia group (D0) and the day 1 (D1), 3 (D3), 5 (D5), 7 (D7), 13 (D13) after exposure to hypoxia (one-way ANOVA followed by LSD multiple comparison test was used and *p* < 0.01). **c** H&E stain of spleen (× 10, bar = 100 μm). **d** Representative figures on IHC staining for CD71 (× 10, bar = 100 μm). **e** The quantification of IHC staining results (wilcox test was used). *P*-value: * *p* < 0.05 and ** *p* < 0.01

To investigate whether the spleen changed during hypoxia exposure, we calculated the spleen index. The spleen index was not influenced by the body weight (Fig. 1a) and index increased significantly on days 3, 5, and 7 compared to the control group. However, after 7 days of hypoxia, the spleen index began to decline and returned to normal by day 13. Furthermore, a significant increase in RBC count was observed from the third day until the end of the experiment (Fig. 1b). To determine which cell populations contributed to splenomegaly, we performed H&E staining (Fig. 1c) and found that the red pulp was enlarged after hypoxia. Furthermore, the CD71 expression via immunohistochemical staining in red pulp significantly increased compared to that of the control animals (Fig. 1d). Accordingly, the CD71 stained area of spleen was significantly expanded by hypoxia intervention, even 24 h after hypoxia exposure (Fig. 1e). However, CD71^+^ cells did not decrease with spleen index.

### Transcriptomic profiling of the spleen at different time points during hypoxia

After characterizing the dynamics of the histological changes, we performed transcriptomic profiling of the spleens on days 1, 3, 5, 7, and 13 of hypoxia exposure. In total, 23 samples collected at 6 different time points were profiled. We abtained 1,239,556,096 total clean reads, with an average of 53,893,743 per sample (Additional Table 2). We selected GRCm38 as our reference genome, and the mapping rate ranged from 94.83 to 95.59%. In the end, 32,775 genes were detected by RNA-seq.

**Fig. 2:**
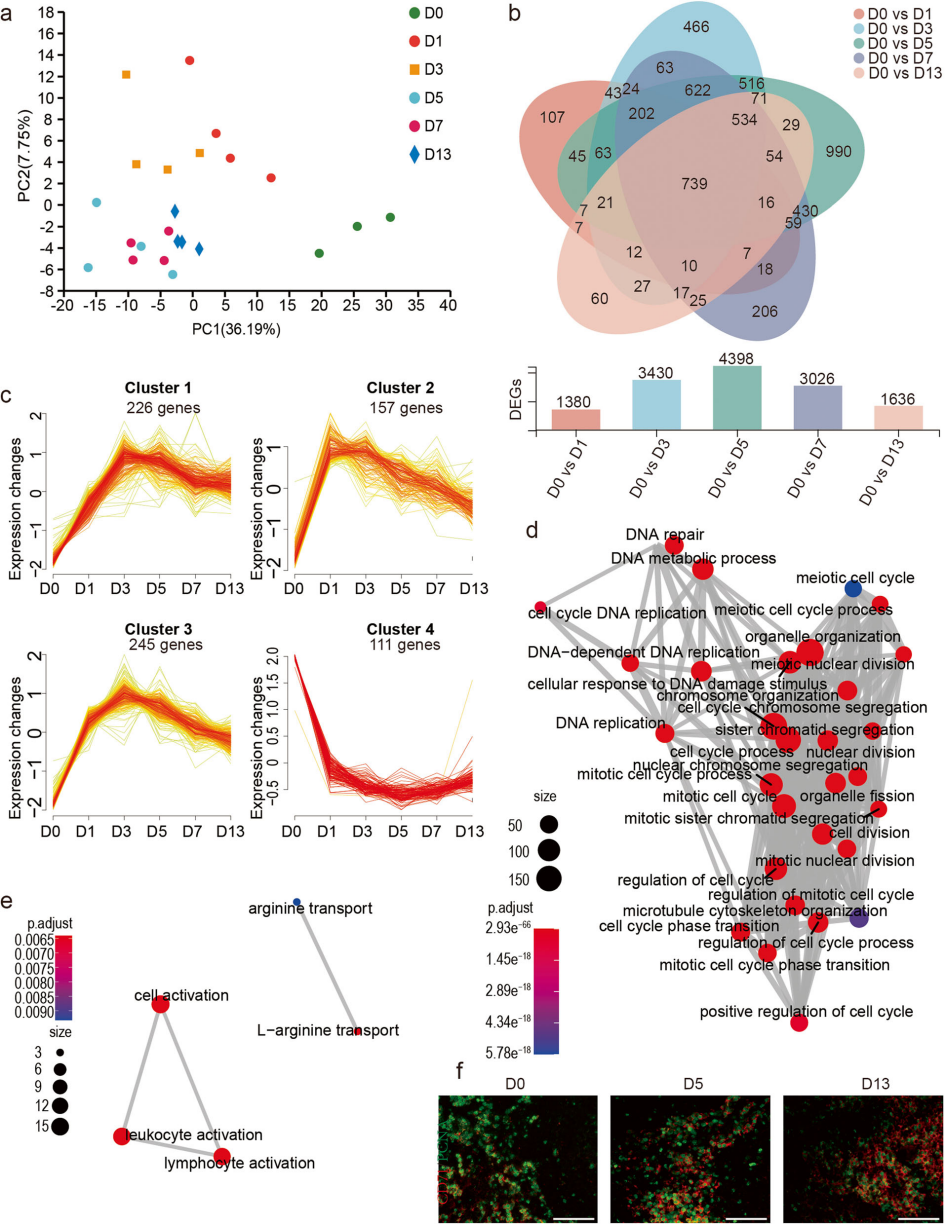
RNA-seq expression profile of spleen exposed to hypoxia. **a** Principal component analysis (PCA) of gene expression. **b** Venn diagram for genes overlapping among five DEG sets (top). The DEGs number in each hypoxia group (bottom). **c** Clusters obtained via the soft clustering method for 739 DEGs of spleen during hypoxia. **d** and **e** Enrichment map of GO terms. Nodes and edges represent GO BP terms and associations between two terms respectively. GO, Gene Ontology; BP, Biological Process; (**d** for cluster 1–3 and **e** for cluster 4 in Fig. 2c). **f** Double immunostaining for PCNA (green) and CD71 (red) on paraffin sections of spleen (× 40, bar = 100 μm)

To examine whether hypoxic stress changed the expression of genes, we conducted principal component analysis (PCA), which revealed higher variation between the Hy and control groups (Fig. 2a). We estimated mean normalized expression values for each gene using RSEM and found that the majority of genes were downregulated by hypoxia exposure (Figure S1a). To detect genes showing differential expression between control and the 5 time points of the Hy group, we performed DESeq2, and identified 1380, 3430, 4398, 3026, and 1636 genes, respectively (Fig. 2b, Additional Tables 3, 4, 5, 6 and 7). Intersection of the DEG datasets identified 739 genes involved in this process (Fig. 2b). These genes play a role mainly in metabolism and the cell cycle based on KEGG analysis (Figure S1b, Additional Tables 8, 9, 10, 11 and 12). To explore the expression pattern of these 739 genes, we performed temporal profile cluster analysis with Mfuzz (Additional Table 13). We found that the expression pattern of 3 modules differed from that of the overall genes (Fig. 2c). Next, we performed GO analysis (Additional Table 14) of these 3 gene modules with upregulated expression and found enrichment for cell proliferation, and cell cycle regulation (Fig. 2d). The other cluster with downregulated genes (cluster 4), was enriched for cell activation, especially of immune cells (Fig. 2e). To identify whether the proliferating cells were erythroid progenitors, we conducted immunofluorescence with anti-CD71 and anti-PCNA antibodies. The results showed that CD71^+^ cells were the main source of cell proliferation (Fig. 2f). Intersection analysis of KEGG pathways indicated that 21 pathways, including Fanconi anemia and the NF-κβ signaling pathways, were common to the five DEG datasets (Additional Figure S1c).

### Key transcription factors during stress erythropoiesis identified by WGCNA analysis

**Fig. 3:**
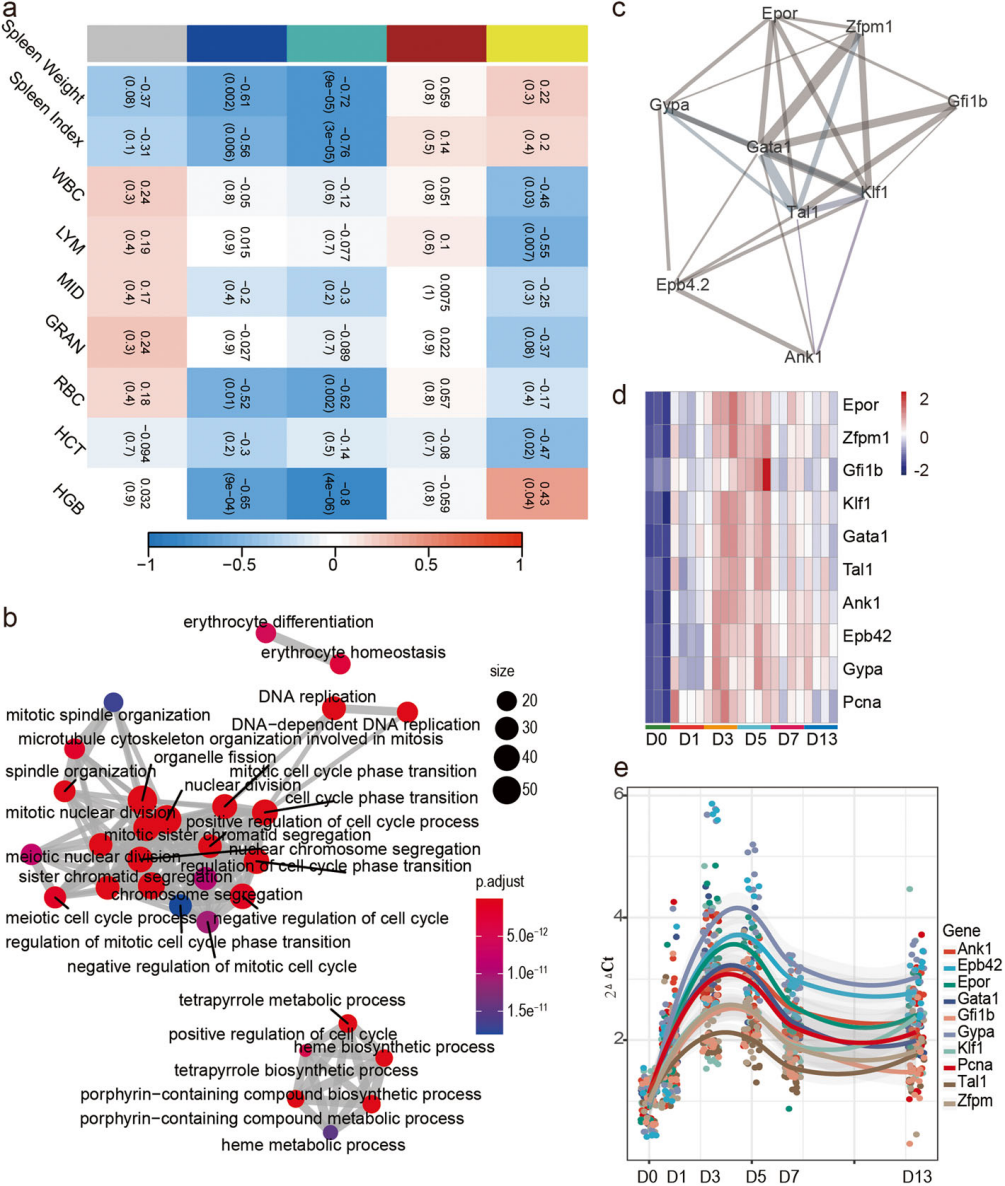
Identification of key module based on WGCNA. **a** Correlation between co-expressed WGCNA module eigengenes and phenotypic traits. Depth of color corresponds to depth of correlation. Positive correlation indicated in red and negative correlation indicated in blue. Significance (P-value) of each module to each external factor presented in parentheses (). **b** Enrichment map of 438 genes. **c** Modules found by MCODE in the network related to erythropoiesis. The edge width was proportional to the score of protein-protein interaction based on the STRING database, The color of edge was weight acquired from WGCNA. **d** The heatmap of erythropoiesis-related gene expression in RNA-seq. **e** qRT-PCR analysis. The mRNA expression levels of 10 selected genes were normalized with the external control gene (*Gapdh*) and were calculated with 2^−ΔΔCt^

**Fig. 4:**
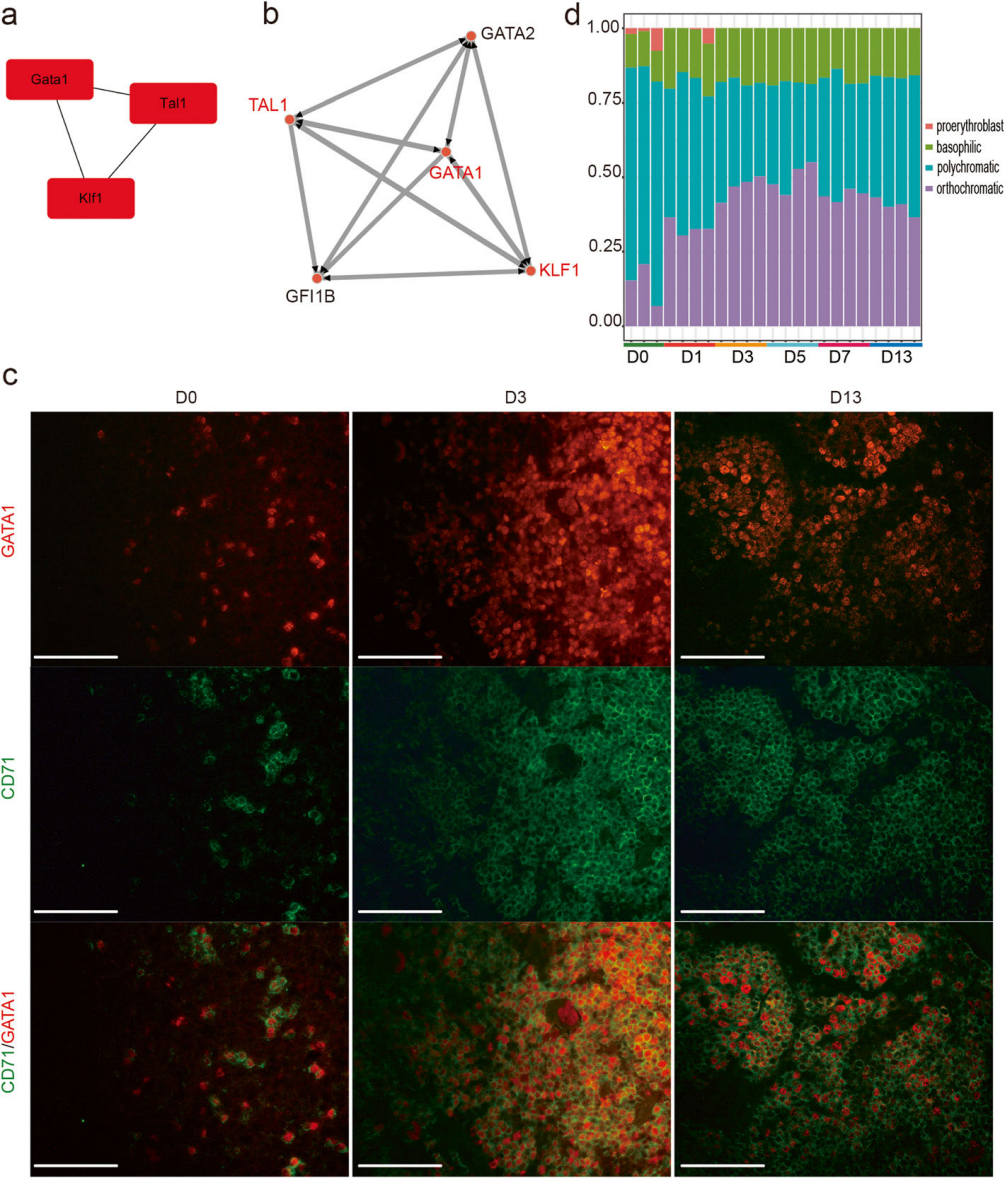
Hub genes were identified in stress erythropoiesis. **a** Results of algorithms from cytoHubba of Cytoscape based on a degree score. **b** The relationship of *Gata1*, *Tal1* and *Klf1* were predicted by ChEA3. **c** Double immunostaining for GATA1 (red) and CD71 (green) on paraffin sections of spleen (× 40, bar = 100 μm). **d** The stacked bar plot of erythroid cells during terminal erythropoiesis was depicted by using CIBERSORT and gene expression file in GSE53983

To identified gene modules associated with increasing erythroid cell numbers, blood counts and spleen index information were extracted, and the correlation between the 5 different color modules was determined by weighted gene co-expression network analysis (WGCNA) (Figure S2, S3a). The module-trait relationship heatmap demonstrated that the blue and turquoise modules were linked to spleen index and RBC counts (Fig. 3a). Turquoise module was the most meaningful module based on its strongly negative correlations with the spleen index and RBC counts (*r* = − 0.76, − 0.62, respectively). To define the kinetics of terminal erythropoiesis in this model, the CIBERSORT analysis was used to achieve the relative fraction of erythroid cells (Fig. 4d, Additional Table 19). The proerythroblasts were rapidly exhausted after exposure to hypoxia (Figure S4a), and the orthochromatic erythroblasts made the extremely contribution during stress erythropoiesis. The WGCNA also showed a great correlation between the Turquoise module and terminal erythropoiesis (Figure S4b). 88 genes in this module were enriched in erythrocyte differentiation GO term, including *Hif1α* (Additional Table 22). Correlations between these genes and *Hif1α* expression levels were calculate. There were 35 genes showed absolute values of Pearson correlation coefficient higher than 0.9 (Figure S5). *Slc4a1, Dyrk3, Fech, Epb42, Rhd* were also in the 739 DEGs. The Arnt (Hif -1β) motif was significantly enriched in in promoter region of these 5 genes (Additional Table 23) by scanning tool FIMO.

There were 438 genes in the intersection of turquoise module and 739 DEGs. The GO enrichment analysis was performed to determine their biological function (Additional Table 16). The analysis showed that 2 Go terms (22 genes) were related to RBC differentiation (Fig. 3b). To explore the interactions within these genes, we performed PPI network analysis by using the STRING database. The network was constructed with 333 genes (nodes) and 5612 gene-gene interactions (edges), adding weight information acquired from WGCNA. MCODE was used to find the module related to RBC differentiation (Fig. 3c, Additional Table 15). The RNA-seq data showed that genes in this module were characterized by high expression on day 3 and 5 after exposure to hypoxia (Fig. 3d). We validated these genes using qPCR (Fig. 3e) and found that the results agreed with the RNA-seq analysis (Figure S3b). The cytoHubba algorithm results applied for hub gene identification showed that *Gata1*, *Tal1*, and *Klf1* played the main role in RBC differentiation (Fig. 4a). Interactions between these three transcription factors were analyzed by ChEA3 (Fig. 4b). Finally, we measured GATA1 expression in the spleen by immunofluorescence and found higher expression, together with CD71 during hypoxia (Fig. 4c).

### Immune response genes are inhibited in the spleen during hypoxia

**Fig. 5:**
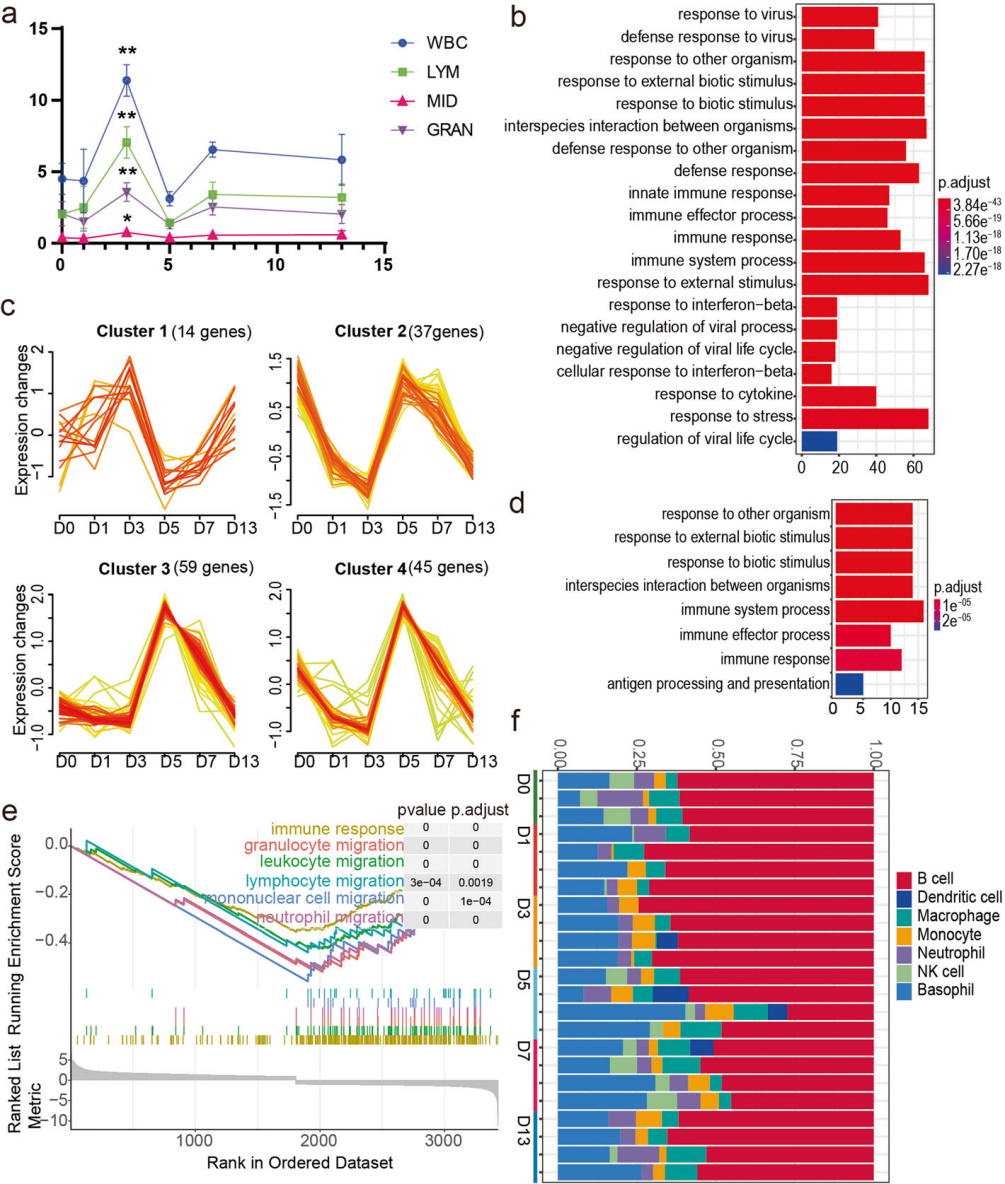
Immune response genes of spleen was inhibited during hypoxia. **a** Line graph of WBC (white blood cell), LYM (lymphocyte cell), MID (monocyte cell) and GRAN (granulocyte cell) counts in peripheral blood (one-way ANOVA followed LSD multiple comparison test was used). P-value: * *p* < 0.05 and ** *p* < 0.01. **b** Bar plot enrichment of GO BP term for yellow module. **c** 4 expression partterns were clustered from yellow module. **d** GO enrichment of cluster 2 in Fig. 5c. **e** GSEA reports for low immune response expression using D3 vs D0 group. **f** The stacked bar plot of different immune cell types was depicted by using CIBERSORT and the spleen specific immune cell gene signature of mice

Genes related to immune cell activation were suppressed after hypoxia exposure (Fig. 2e). Another interesting finding was that immune cells, such as white blood cells (WBC), only increased significantly on day 3 (Fig. 5a). To identify genes involved in this process, we found that genes in the yellow module was negativity relate to the white blood cell and lymphocyte cell in peripheral blood (Fig. 3a). These genes also enriched in the immune response (Fig. 5b). Next, we clustered these genes to 4 patterns and found that expression of 37 genes in Cluster 2 decreased rapidly in 3 days (Fig. 5c, Additional Table 17). Biological function enrichment analysis also showed immune response (Fig. 5d). Furthermore, based on the GSEA analysis, we found that genes related to immune cell migration were downregulated on day 3 (Fig. 5e, Additional Table 18). To investigate changes in immune cell types in the spleen, we used the CIBERSORT analytical tool (Fig. 5f, Additional Table 20). The result showed that B cells were the main component, and that they increased slightly on days 1 and 3 of hypoxia exposure (Kruskal-Wallis test, *p* = 0.047, 0.047). NK cells decreased rapidly on days 1, 3, and 13 (Kruskal-Wallis test, *p* = 0.01, 0.01 and 0.01). However. other cell types did not change during hypoxic stress.

## Discussion

Our data showed that stress erythropoiesis occurs in the spleen to compensate for the reduced oxygen supply during hypoxia, resulting in splenomegaly, especially during the first week. Transcriptomic analysis showed that hypoxia promotes splenic cell proliferation and represses immune cell activation. Furthermore, *Gata1*, *Tal1*, and *Klf1* were identified as key TFs regulating stress erythropoiesis in the spleen. In silico analysis of immune cell populations demonstrated inhibition of the immune response. Transcriptomic analysis of global gene expression patterns during hypoxia sheds new light on how the spleen adapts to this stress.

Splenomegaly can be caused by three main factors: increased splenic function, infiltration, or congestion [29]. In this case, the spleen enlarged due to the expansion of CD71^+^ cells during the first week. It is widely believed that CD71 expression is very high in early erythroid precursors [6]. Moreover, flow cytometry analysis has shown that cell-surface CD71 on EryA peaks on day 3 of hypoxia [30]. This means that splenomegaly was caused by increased splenic function during stress erythropoiesis.

Hypoxia changes energy metabolism, mitochondrial respiration, lipid and carbon metabolism, as well as nutrient acquisition by the cell [31], and also plays an important role in maintaining the proliferation of stem cells [32]. We found that the Fanconi anemia pathway, which promotes stem cell function and survival [33], was enriched in each of the DEG datasets. EPO promotes the viability, proliferation, and terminal differentiation of erythroid precursors, is also known to inhibit inflammation by decreasing NF-κβ signaling [34, 35], which is at the center of the molecular mechanisms controlling inflammation [36, 37]. Genes in this pathway were mainly downregulated (Figure S4c). The “canonical” pathway of NF-κβ activation is triggered by proinflammatory cytokines, such as IL-1β. In vitro experiments have determined that increased levels of IL-1β can enhance the proliferation of stress erythroid progenitors [26, 27]. However, in our dataset, *IL-1β* was downregulated with the expansion of erythroid progenitors. The NF-κβ signaling pathway may be key pathway regulating stress erythropoiesis and the immune response.

*Gata1, Klf1,* and *Tal1* regulate erythroid differentiation, and mice deficient in these TFs show severe embryonic lethality [38–42]. GATA1 targets genes involved in heme biosynthesis, erythropoietin signaling, and anti-apoptotic and proliferation pathways, and is also required for *Epor* expression [43, 44]. GATA1 also modulates the transcription of *Tal1* and *Klf1* during erythroid differentiation [45, 46]. Ex vivo studies showed that these three TFs (GATA1, FLI1, TAL1) play a predominant role, above that of cytokines (EPO or TPO), in the capacity of bipotent populations (BPPs) to differentiate into erythroblasts (ERYs) or megakaryocytes (MKs), and that ERYs were biased toward expression of GT (GATA1, TAL1) [47]. On the other hand, *Klf1* upregulation occurs along the E-MEP trajectory and promoted the lineage specification to erythroid differentiation [48, 49]. The forecasted relative fraction of cell type in terminal erythropoiesis stage showed the ratio of orthochromatic erythroblast cells increased with hypoxia exposure, while *Gata1*, *Tal1* and *Klf1* were down-regulated during terminal erythroid differentiation [50, 51]. Increasing co-expression of *Gata1*, *Tal1* and *Klf1* not only influence MEP cell fate but can also enlarge the pool of CD71^+^ cells to increase EPO sensitivity.

It has been shown that *Tal1* expression blocks T cell differentiation [52]. Unfortunately, we failed to estimate T cells in the spleen. Hypoxia plays an important role in innate immunity. NK cells, a subset of innate lymphocytes, are sensitive to conditions of hypoxia [53, 54]. Our results are in agreement with a hypoxia–ischemia model that showed that NK cells expression was reduced in the spleen [55]. However, another study showed that when rats were exposed to mild hypoxia, the NK cell ratio was significantly higher, but then decreased after 7 days [56]. Increasing evidence demonstrates that hypoxia regulates multiple immune processes, such as cell migration, antigen presentation and immune effector functions. Transcriptomic analysis of the large yellow croaker also showed that immune response genes were downregulated in spleen [57]. This inconsistency with the phenotypic values that leukocytes were transiently increased on day 3. Other studies on rats and fishes suggested that hypoxia stress increased WBC number in peripheral blood [58–60]. The evidence of transcriptome and estimated immune cells excluded the involving of spleen in this process, other immune organs might contribute it. During short-term hypoxia, the spleen downregulated immune-related genes and reduced some kinds of immune cells to compensate stress erythropoiesis.

Although, we conducted the CIBERSORT analysis to find out the fraction of erythroid and immune cells, the relative ratio of these two kinds of cells is still unknown. This makes it difficult to separate the effects of altered gene expression from the effects of changing cell type proportions in this study and single cell RNA-seq may be required for the future studies and help address this issue.

It is known that hypoxia-inducible factor (HIF), a key transcriptional effector of the hypoxia response, facilitates a high production of red blood cell. Each functional HIF unit is composed of constitutively expressed β subunit (HIF-1β) and an oxygen responsive α subunit (HIF-1α, 2α or 3α). However, only *Hif3α*, involved in the regulation of EPO signaling [61], was upregulated in our data. Two variants of the *HIF3A* gene were associated with familial erythrocytosis in human [62]. During the hypoxic condition, stabilized HIF-α activates transcription of target genes with Arnt (HIF-1β) in the nucleus [63]. *Slc4a1*, *Dyrk3*, *Fech*, *Epb42*, *Rhd* contained Arnt motif in promotor region and the expression of these genes are likely regulated by Hif3α. It is important to note that *Dyrk3* is erythroid-restricted gene and act to attenuate erythroblast development [64, 65]. According to the expression pattern of *Dyrk3* and *Hif3α* (Additional Table 13), *Hif3α* increased rapidly in response to hypoxia and might promote transcription of down-stream genes *Dyrk3,* to place an upper limit on red cell production during stress erythropoiesis. Further studies on the function of *Hif3α* in stress erythropoiesis are still needed.

As was previously reported, Erythropoietin and BMP4-dependent stress erythropoiesis are two ways to regulate erythrocyte differentiation. However, *Bmp4* expression only significantly downregulated on day 5 and 7 in our data, *Epor* was continuously upregulated during hypoxia. This means that although anemia and hypoxia both produce tissue hypoxia, the strategies of the spleen to overcome this stress conditions are different.

## Conclusions

In this study, we reported the global change of splenic gene expression by time-series RNA-seq during hypoxia treatment. The spleen enlarged with red pulp to generate more erythrocytes to conquer this stress. *Gata1*, *Tal1* and *Klf1* were major TFs to maintain cell proliferation and stress erythropoiesis. Both gene expression patterns and GSEA analysis showed immune response genes was inhibited, and NK cells forecasted in silico decreased during hypoxia. The NF-κβ signaling pathway, always functional during hypoxia, may be as a key pathway regulating both stress erythropoiesis and immune response. Additionally, *Hif3α* might involve in this process. At last, this study will provide the availability data of spleen at different time exposure to hypoxia.

## Methods

### Mice

All animal procedures were conducted in accordance with the Guide for the Care and Use of Laboratory Animals and were approved by the Animal Welfare and Ethic Committee of the Northwest Institute, Chinese Academy of Sciences. ICR mice (7–8 weeks old) were purchased from the *SIBEIFU* company. We only used male mice to exclude any effects of the estrous cycle on erythropoiesis. We randomly assigned the mice to two groups: 3 in the normoxic (Nox) group and 20 in the hypoxic (Hy) group. Animals in the Hy group were placed in an altitude chamber with a pressure of 52.93 KPa, corresponding to an altitude of 5000 m. The concentration of oxygen in the chamber was 19.5%. It was opened daily for 1 h to provide the animals with fresh water, food and straw. Animals stayed in the chamber for the duration of the experiment. We analyzed 5 time points during hypoxic exposure: on days 1, 3, 5, 7 and 13. Animals in the control (Nox group) were sacrificed upon arrival at Xining, and the body weight and the weight of the spleen were recorded. The effects of hypoxia on the spleen were investigated based on the spleen index, calculated as the weight of the spleen (in mg)/body weight (in g). The body weight index was calculated as follows: body weight on a specific day of hypoxia exposure /the mean weight of the control.

### Blood counts

Fresh blood was collected from anesthetized mice in EDTA tubes by retro-orbital bleeding, and was used to determine RBC, hematocrit, and hemoglobin values using a fully automated hematology analyzer (PROKAN, PE-6800VET, China).

### Immunohistochemistry (IHC) and immunofluorescence (IF)

Spleens were fixed overnight with 4% paraformaldehyde (PFA). After dehydration in ethanol, samples were embedded in paraffin (HistoCore Arcadia, *Leica*, Mannheim, Germany). Paraffin-embedded tissues were cut into 4 μm slices with a microtome (Leica RM2235, *Leica*, Mannheim, Germany). After deparaffinization and rehydration, sections were stained with hematoxylin and eosin (H&E). For IHC, the sections were boiled in 10 mM sodium citrate buffer (pH 6.0) for 20 min and washed with 0.01 M phosphate-buffered saline (PBS) for 5 min. This was repeated 3 times at room temperature (RT). Endogenous peroxidase activity was blocked with 3% H_2_O_2_ for 10 min at RT. Sections were washed 3 times with PBS (5 min per wash) and incubated for 30 min at 37° with 10% normal goat serum. Next, samples were incubated overnight at 4 °C with anti-CD71 (ab84036, *Abcam*) antibody diluted in 3% BSA (1:200). Sections were then washed with PBS, followed by incubation for 30 min at 37° with HRP-conjugated goat anti-rabbit secondary antibody (Servicebio*;* Wuhan, China). After 3 washes with PBS (10 min per wash), antigens were visualized by adding 3,3-diaminobenzidine (DAB, ZSGB-BIO, Beijing, China) and sections were counterstained with Ehrlich’s hematoxylin. Slides were examined with a microscope (ECLIPSE E200, *Nikon*, Tokyo, Japan), and images were captured by CCD (MS60, *MshOt*, Guangzhou, China).

Immunofluorescent staining procedure: following antigen retrieval, the tissue sections were incubated for 5 min with a spontaneous fluorescence quenching reagent (Wuhan Servicebio Technology Co., Ltd., Wuhan, China). Sections were then incubated for 1 h at RT with 10% normal donkey serum. Next, the samples were incubated overnight at 4° with the primary antibodies (diluted 1:200): anti-CD71 (ab84036, *Abcam*), anti-GATA1 (sc-265, *Santa* Cruz) and anti-PCNA (RLM3031, *Ruiying Biotechnology*, China). Sections were then washed and incubated with the secondary antibodies for 2 h at RT. After 3 final washes with PBS (10 min each), the sections were stained with Hoechst33342 (H33342) (*Sigma*, St. Louis, MO, USA) and mounted with 50% glycerol for microscopic examination (*Leica*, Mannheim, Germany).

### Quantitative analysis of immunohistochemical tissue sections

Fiji (version 1.52 g) was used to determine the proportion of tissue that was CD71 positive. This proportion was estimated by calculating the total area of the spleen (defined by hematoxylin staining) based on 10–24 serial sections. The relative level of CD71 positivity in the tissue sections was calculated as follows: positive area / total area of the spleen.

### RNA isolation, cDNA library construction and sequencing

Total RNA was extracted from the 23 spleens by using TRIzol® reagent, according to the manufacturer’s instructions (*Invitrogen*, CA) and genomic DNA was removed with DNase I (*TaKara*). RNA quality was determined using a 2100 Bioanalyser (Agilent) and quantity using a ND-2000 system (*NanoDrop* Technologies). Only high-quality RNA samples (OD260/280 = 1.8 ~ 2.2, OD260/230 ≥ 2.0, RIN ≥ 6.5, 28S:18S ≥ 1.0, > 2 μg) were used to construct the sequencing library..

The RNA-seq library for transcriptome analysis was prepared using the TruSeq™ RNA sample preparation kit (*San Diego*, CA) and 1 μg of total RNA per sample. Briefly, mRNA was isolated by the polyA selection method with oligo (dT) beads and then treated with fragmentation buffer. Next, double-stranded cDNA was synthesized using a SuperScript double-stranded cDNA synthesis kit (*Invitrogen*, CA) and random hexamer primers (Illumina). The synthesized cDNA was subjected to end-repair, phosphorylation, and ‘A’ base addition according to Illumina’s library construction protocol. Libraries were size-selected for cDNA fragments of 200–300 bp by means of 2% low range ultra-agarose, followed by PCR amplification (15 PCR cycles) using Phusion DNA polymerase (NEB). After quantification with a TBS380 mini-fluorometer, the paired-end RNA-seq library was sequenced using the Illumina NovaSeq 6000 platform (2 × 150 bp read length).

### RNA-Seq analysis

The raw paired-end reads were trimmed and quality controlled by SeqPrep (https://github.com/jstjohn/SeqPrep) and Sickle (https://github.com/najoshi/sickle) with default parameters. The clean reads were separately aligned to the reference genome (GRCm38, http://asia.ensembl.org/Mus_musculus/Info/Index) with orientation mode using TopHat (http://tophat.cbcb.umd.edu/, version2.0.0) [66] software. We acquired 185.02 Gb of clean data. The alignment rate was above 94% across all samples. The expression level of each transcript was calculated Transcripts Per Kilobase of exon model per Million mapped reads (TPM) by using RSEM [67] (http://deweylab.biostat.wisc.edu/rsem/). To identify differentially expressed genes (DEGs) between the Nox and Hy groups, EdgeR [68] was utilized to calculate the fold change in gene expression. DEGs were selected based on an adjusted *P*-value < 0.05 and |log2FoldChange| > =1. In addition, functional-enrichment analysis, including GO and KEGG (www.kegg.jp/kegg/kegg1.html) [69], was performed using the R package clusterProfiler [70] (version 3.17.3). Significantly overrepresented biological process GO terms were identified based on a q-value < 0.01. We identified which DEGs were significantly enriched in GO terms when compared with the whole-transcriptomic background.

### Time-series clustering of differentially expressed genes

The R package Mfuzz (v2.49) [71] was used for clustering analysis of the intersection of DEGs along time. Mfuzz (https://bioconductor.org/packages/release/bioc/html/Mfuzz.html) is a software package for soft-clustering of microarray data which operates based on fuzzy c-means algorithm. Average expression values at each time point were used as the input to generate 4 (k = 4) clusters based on the expression trend.

### Weighted gene co-expression network analysis (WGNCA)

To perform unsigned WGCNA analysis, we used the R WGCNA package (https://cran.r-project.org/package=WGCNA) [72]. (Soft-power 5, mergeCutheight 0.25, minModuleSize 30). We identified 5 modules. Module-Trait relationships were calculated by Pearson correlation between the eigengene of each module and the specific phenotype data. Module eigengenes and orthogroup connectivity were calculated separately in each network using the moduleEigengenes() and intramodularConnectivity() functions in WGCNA, respectively.

Protein–protein interaction (PPI) networks can assist in the identification of key genes and pivotal gene modules involved in the response to hypoxia. Relations between genes were visualized by means of Cytoscape (v3.8.0) [73]; MCODE verified the key modules, and the hub gene were identified with cytoHubba. To explore the relationship among three transcription factors (TFs), we used ChEA3 [74]. ChEA3 is a database that performs TFs enrichment analysis based on ChIP-seq experiments.

### Predicted immune cells

The murine spleen-specific expression matrix by ImmuCC [75] was used for analysis. Raw RNA-seq read counts were normalized and processed with CIBERSORT (https://github.com/jason-weirather/CIBERSORT) [76] with no quantile normalization and 1000 permutations as parameters. All samples were run to quantify the relative proportions of 7 immune cell types.

The matrix of gene expressions of GSE53983 [77] was used in CIBERSORT analysis (permutation = 1000) to estimate the relative fraction of proerythroblast, basophilic, polychromatic, and orthochromatic erythroid cells in terminal erythropoiesis stage.

### FIMO motif analysis

Analysis was performed at the FIMO [78] website: http://meme.nbcr.net/meme/tools/fimo. using a *p*-value output threshold of 0.001 and motif (MA0004.1) information was obtained from the JASPAR database (http://jaspar.genereg.net/). The promotor sequences (about 2 kB region upstream of transcription start site) were downloaded from NCBI genome browser.

### qRT-PCR analysis

Total RNA was extracted with Trizol (Ambion, Austin, TX, USA), according to the manufacturer’s instructions. The cDNAs were synthesized using Honor™ II 1st Strand Cdna Synthesis SuperMix (*Novogene*, China). The qRT-PCR analysis was performed using the ABI ViiA7 Real-time PCR System (Applied Biosystems, Foster City, CA, USA) and SYBR Green master mix (*Genstar*, Guangzhou, China). The primer sequences are listed in Additional Table 1. PCR conditions were: 15 min at 95 °C and 40 cycles of 95 °C for 20 s and 60 °C for 1 min.

### Statistical analysis

Statistical analysis was performed using either by R project or GraphPad Prism 8. Results are expressed as the mean ± standard deviation, unless otherwise indicated. The Shapiro-Wilk normality test was used to analyze whether the continuous variables conformed to a normal distribution. Comparison between multiple groups (between the 5 Hy groups and the Nox group) was performed by one-way ANOVA for data with normal distribution and with Kruskal-Wallis or Wilcox tests for data with non-normal distribution. A *P* value of less than 0.05 was considered significant. The correlation between genes was calculated by using the Pearson correlation coefficient. Cor.full() function in “tinyarray” package (https://github.com/xjsun1221/tinyarray) was used. Data visualization was performed using R version 4.0.2 and packages: “ggplot2” (https://github.com/tidyverse/ggplot2) [79], “ggsci” (https://CRAN.R-project.org/package=ggsci), “ggpubr” (https://cran.r-project.org/web/packages/ggpubr/index.htm), “ggsignif” (https://CRAN.R-project.org/package=ggsignif), and “Pheatmap” (https://cran.r-project.org/web/packages/pheatmap/index.html). Venn diagrams were constructed with TBtools [80].

## Supplementary Information

**Additional file 1: Figure S1.** (a) The density distribution map of transcript per million (TPM). (b) The bubble chart showing the KEGG pathways of 739 DEGs. (c) Venn diagram illustrating the overlapped KEGG pathways. (d) KEGG pathway annotation of intersection and specific parts in Figure S1c. (The color represented each comparison groups are same with Figure S1c). **Figure S2.** (a) Power value for the adjacency matrix in WGCNA, where the red line signals 0.85 on the vertical axis. (b) The mean connectivity of WGCNA analysis. **Figure S3.** (a) Hierarchical cluster tree showing coexpression modules identified by WGCNA. (b) Validation of the transcriptome data by qRT-PCR. (r were calculated by Pearson correlation). **Figure S4.** (a) The relative ratio of erythroid cells during terminal erythropoiesis stage. (Kruskal-Wallis test was used and *P* value were corrected by bonferroni) (b) Module-trait relationships plot. Each row corresponds to a module, column to different cell types during terminal erythropoiesis. (c) Heatmap of genes involved in the NF-kappa B signaling pathway. **Figure S5.** The correlation between erythrocyte differentiation related genes in turquoise module with *Hif1a*.

**Additional file 2: Additional Table 1.** Specific primers of genes for qRT-PCR. **Additional Table 2.** Summary of QC for the time-series RNA-seq. **Additional Table 3.** Differentially expressed genes between D1 and D0. **Additional Table 4.** Differentially expressed genes between D3 and D0. **Additional Table 5.** Differentially expressed genes between D5 and D0. **Additional Table 6.** Differentially expressed genes between D7 and D0. **Additional Table 7.** Differentially expressed genes between D13 and D0. **Additional Table 8.** Results of KEGG pathway enrichment analysis for DEGs between D1 and D0. **Additional Table 9.** Results of KEGG pathway enrichment analysis for DEGs between D3 and D0. **Additional Table 10.** Results of KEGG pathway enrichment analysis for DEGs between D5 and D0. **Additional Table 11.** Results of KEGG pathway enrichment analysis for DEGs between D7 and D0. **Additional Table 12.** Results of KEGG pathway enrichment analysis for DEGs between D13 and D0. **Additional Table 13.** The clusters of intersection DEGs (739 genes) in Fig. 2b by Muffz analysis. (related to Fig. 2c). **Additional Table 14.** GO enrichment analysis results of 739 genes. (Red for unregulated genes, blue for downregulated genes). **Additional Table 15.** Edge information of key module regulating the stress erythropoiesis. (related to Fig. 3c). **Additional Table 16.** GO enrichment analysis for intersection of turquoise module and 739 DEGs. (related to Fig. 3b). **Additional Table 17.** The clusters of genes in yellow module by Muffz analysis. (related to Fig. 5c). **Additional Table 18.** Results of GSEA for DEGs between D3 and D0. (related to Fig. 5e). **Additional Table 19.** The results of CIBERSORT analysis to estimate the relative fraction of cells during terminal erythropoiesis stage. (related to Fig. 4d). **Additional Table 20.** The results of CIBERSORT analysis to estimate the relative fraction of immune cells. (related to Fig. 5f and Figure S4a). **Additional Table 21.** The correlation between genes with Hif1a (Absolute value of the correlation coefficient > 0.9). (related to Figure S5). **Additional Table 22.** The weight of erythrocyte differentiation related genes in turquoise module. (related to Figure S5). **Additional Table 23.** Scanning for occurrences of Arnt motif in promotor region of 5 genes.

## Data Availability

Raw sequence data are accessible at NCBI under the BioProject accession number PRJNA705739 on reasonable request.

## Abbreviations

WP: White pulp
RP: Red pulp
RBC: Red blood cell
HSCs: Hematopoietic stem cells
EPO: Erythropoietin
BMP4: Bone morphogenetic protein 4
TFs: Transcription factors
Nox group: Normoxic group
Hy group: Hypoxic group

## References

[1] Mebius RE, Kraal G (2005). Structure and function of the spleen. Nat Rev Immunol.

[2] Lewis SM, Williams A, Eisenbarth SC (2019). Structure-function of the immune system in the spleen. Sci Immunol.

[3] Lauda E, Haam E (1931). Importance of the spleen as a reservoir for red blood cells. Exp Biol Med.

[4] Potocnik S, Wintour E (1996). Development of the spleen as a red blood cell reservoir in lambs. Reprod Fertil Dev.

[5] Short C, Lim HK, Tan J, O’Neill HC (2019). Targeting the spleen as an alternative site for hematopoiesis. BioEssays..

[6] Socolovsky M (2007). Molecular insights into stress erythropoiesis. Curr Opin Hematol.

[7] Inra CN, Zhou BO, Acar M, Murphy MM, Richardson J, Zhao Z, Morrison SJ (2015). A perisinusoidal niche for extramedullary haematopoiesis in the spleen. Nature..

[8] Sonmez G, Ozturk E, Basekim CC, Mutlu H, Kilic S, Onem Y, Kizilkaya E (2007). Effects of altitude on spleen volume: Sonographic assessment. J Clin Ultrasound.

[9] Purdy GM, James MA, Rees JL, Ondrus P, Keess JL, Day TA, Steinback CD (2019). Spleen reactivity during incremental ascent to altitude. J Appl Physiol.

[10] Holmström P, Mulder E, Starfelt V, Lodin-Sundström A, Schagatay E (2020). Spleen size and function in Sherpa living high, Sherpa Living Low and Nepalese Lowlanders. Front Physiol.

[11] Schagatay E, Holmström P, Mulder E, Limbu P, Schagatay FS, Engan H, Lodin-Sundström A (2020). Spleen volume and contraction during apnea in Mt. Everest climbers and Everest Base camp trekkers. High Alt Med Biol.

[12] Cook SF, Alafi MH (1956). Role of the spleen in acclimatization to hypoxia. Am J Physiology-Legacy Content.

[13] Clegg EJ (1983). Morphometric studies of the spleen of the hypoxic mouse. J Microsc.

[14] Dalton AJ, Jones BF. Organ Changes in Rats Exposed Repeatedly to Lowered Oxygen Tension with Reduced Barometric Pressure. J Natl Cancer Inst. 1945. 10.1093/jnci/6.3.161. 21009554

[15] Turner MS, Hurst JM, Yoffey JM (1967). Viii. Effect of hypoxia and post-hypoxic polycythaemia (rebound) on mouse marrow and spleen. Br J Haematol.

[16] Stutte HJ, Sakuma T, Falk S, Schneider M (1986). Splenic erythropoiesis in rats under hypoxic and post-hypoxic conditions. Vichows Archiv A Pathol Anat.

[17] Rapp JP, Christian JJ (1963). Splenic Extramedullary hematopoiesis in grouped male mice. Exp Biol Med.

[18] Paulson RF, Hariharan S, Little JA (2020). Stress erythropoiesis: definitions and models for its study. Exp Hematol.

[19] Harada T, Tsuboi I, Hirabayashi Y, Kosaku K, Naito M, Hara H, Inoue T, Aizawa S (2015). Decreased “ineffective erythropoiesis” preserves polycythemia in mice under long-term hypoxia. Clin Exp Med.

[20] Millot S, Andrieu V, Letteron P, Lyoumi S, Hurtado-Nedelec M, Karim Z (2010). Erythropoietin stimulates spleen BMP4-dependent stress erythropoiesis and partially corrects anemia in a mouse model of generalized inflammation. Blood..

[21] Lenox LE, Perry JM, Paulson RF (2005). BMP4 and Madh5 regulate the erythroid response to acute anemia. Blood..

[22] Perry JM, Harandi OF, Porayette P, Hegde S, Kannan AK, Paulson RF (2009). Maintenance of the BMP4-dependent stress erythropoiesis pathway in the murine spleen requires hedgehog signaling. Blood..

[23] Perry JM, Harandi OF, Paulson RF (2007). BMP4, SCF, and hypoxia cooperatively regulate the expansion of murine stress erythroid progenitors. Blood..

[24] Wu D-C, Paulson RF (2010). Hypoxia regulates BMP4 expression in the murine spleen during the recovery from acute Anemia. PLoS One.

[25] Palazon A, Goldrath AW, Nizet V, Johnson RS (2014). HIF transcription factors, inflammation, and immunity. Immunity..

[26] Bennett LF, Liao C, Quickel MD, Yeoh BS, Vijay-Kumar M, Hankey-Giblin P, Prabhu KS, Paulson RF (2019). Inflammation induces stress erythropoiesis through heme-dependent activation of SPI-C. Sci Signal.

[27] Paulson RF, Ruan B, Hao S, Chen Y (2020). Stress erythropoiesis is a key inflammatory response. Cells..

[28] Morceau F, Dicato M, Diederich M. Pro-inflammatory cytokine-mediated Anemia: regarding molecular mechanisms of erythropoiesis. Mediat Inflamm. 2009;2009. 10.1155/2009/405016. 10.1155/2009/405016 PMC2830572 20204172

[29] McKenzie CV, Colonne CK, Yeo JH, Fraser ST (2018). Splenomegaly: pathophysiological bases and therapeutic options. Int J Biochem Cell Biol.

[30] Porpiglia E, Hidalgo D, Koulnis M, Tzafriri AR, Socolovsky M. Stat5 signaling specifies basal versus stress Erythropoietic responses through distinct binary and graded dynamic modalities. PLoS Biol. 2012;10(8). 10.1371/journal.pbio.1001383. 10.1371/journal.pbio.1001383 PMC3433736 22969412

[31] Lee P, Chandel NS, Celeste SM (2020). Cellular adaptation to hypoxia through HIFs and beyond. Nat Rev Mol Cell Biol.

[32] Nekanti U, Dastidar S, Venugopal P, Totey S, Ta M (2010). Increased proliferation and analysis of differential gene expression in human Wharton’s jelly-derived Mesenchymal stromal cells under hypoxia. Int J Biol Sci.

[33] Bagby G. Recent advances in understanding hematopoiesis in Fanconi Anemia. F1000Res. 2018;7. 10.12688/f1000research.13213.1. 10.12688/f1000research.13213.1 PMC5785713 29399332

[34] Nairz M, Schroll A, Moschen AR, Sonnweber T, Theurl M, Theurl I, Taub N, Jamnig C, Neurauter D, Huber LA, Tilg H, Moser PL, Weiss G (2011). Erythropoietin contrastingly affects bacterial infection and experimental colitis by inhibiting nuclear factor-κB-inducible immune pathways. Immunity..

[35] Bunn HF (2013). Erythropoietin. Cold Spring Harbor Perspect Med.

[36] Rothschild DE, McDaniel DK, Ringel-Scaia VM, Allen IC (2018). Modulating inflammation through the negative regulation of NF-κB signaling. J Leukoc Biol.

[37] Liu T, Zhang L, Joo D, Sun S-C (2017). NF-κB signaling in inflammation. Sig Transduct Target Ther.

[38] Fujiwara Y, Browne CP, Cunniff K, Goff SC, Orkin SH (1996). Arrested development of embryonic red cell precursors in mouse embryos lacking transcription factor GATA-1. Proc Natl Acad Sci U S A.

[39] Whyatt D, Lindeboom F, Karis A, Ferreira R, Milot E, Hendriks R, de Bruijn M, Langeveld A, Gribnau J, Grosveld F, Philipsen S (2000). An intrinsic but cell-nonautonomous defect in GATA-1-overexpressing mouse erythroid cells. Nature..

[40] Nuez B, Michalovich D, Bygrave A, Ploemacher R, Grosveld F (1995). Defective haematopoiesis in fetal liver resulting from inactivation of the EKLF gene. Nature..

[41] Perkins AC, Sharpe AH, Orkin SH (1995). Lethal beta-thalassaemia in mice lacking the erythroid CACCC-transcription factor EKLF. Nature..

[42] Shivdasani RA, Mayer EL, Orkin SH (1995). Absence of blood formation in mice lacking the T-cell leukaemia oncoprotein tal-1/SCL. Nature..

[43] Zon LI, Youssoufian H, Mather C, Lodish HF, Orkin SH (1991). Activation of the erythropoietin receptor promoter by transcription factor GATA-1. Proc Natl Acad Sci U S A.

[44] Welch JJ, Watts JA, Vakoc CR, Yao Y, Wang H, Hardison RC, Blobel GA, Chodosh LA, Weiss MJ (2004). Global regulation of erythroid gene expression by transcription factor GATA-1. Blood..

[45] Anderson KP, Crable SC, Lingrel JB (2000). The GATA-E box-GATA motif in the EKLF promoter is required for in vivo expression. Blood..

[46] Han GC, Vinayachandran V, Bataille AR, Park B, Chan-Salis KY, Keller CA (2015). Genome-wide organization of GATA1 and TAL1 determined at high resolution. Mol Cell Biol.

[47] Dalby A, Ballester-Beltrán J, Lincetto C, Mueller A, Foad N, Evans A, Baye J, Turro E, Moreau T, Tijssen MR, Ghevaert C (2018). Transcription factor levels after forward programming of human pluripotent stem cells with GATA1, FLI1, and TAL1 determine megakaryocyte versus Erythroid cell fate decision. Stem Cell Reports.

[48] Psaila B, Barkas N, Iskander D, Roy A, Anderson S, Ashley N, Caputo VS, Lichtenberg J, Loaiza S, Bodine DM, Karadimitris A, Mead AJ, Roberts I (2016). Single-cell profiling of human megakaryocyte-erythroid progenitors identifies distinct megakaryocyte and erythroid differentiation pathways. Genome Biol.

[49] Siatecka M, Bieker JJ (2011). The multifunctional role of EKLF/KLF1 during erythropoiesis. Blood..

[50] Huang P, Zhao Y, Zhong J, Zhang X, Liu Q, Qiu X, Chen S, Yan H, Hillyer C, Mohandas N, Pan X, Xu X (2020). Putative regulators for the continuum of erythroid differentiation revealed by single-cell transcriptome of human BM and UCB cells. PNAS..

[51] Gautier E-F, Leduc M, Ladli M, Schulz VP, Lefèvre C, Boussaid I, Fontenay M, Lacombe C, Verdier F, Guillonneau F, Hillyer CD, Mohandas N, Gallagher PG, Mayeux P (2020). Comprehensive proteomic analysis of murine terminal erythroid differentiation. Blood Advances.

[52] Palii CG, Perez-Iratxeta C, Yao Z, Cao Y, Dai F, Davison J, Atkins H, Allan D, Dilworth FJ, Gentleman R, Tapscott SJ, Brand M (2011). Differential genomic targeting of the transcription factor TAL1 in alternate haematopoietic lineages. EMBO J.

[53] Parodi M, Raggi F, Cangelosi D, Manzini C, Balsamo M, Blengio F, et al. Hypoxia modifies the Transcriptome of human NK cells, modulates their Immunoregulatory profile, and influences NK cell subset migration. Front Immunol. 2018;9. 10.3389/fimmu.2018.02358. 10.3389/fimmu.2018.02358 PMC6232835 30459756

[54] Velásquez SY, Killian D, Schulte J, Sticht C, Thiel M, Lindner HA (2016). Short term hypoxia synergizes with interleukin 15 priming in driving glycolytic gene transcription and supports human natural killer cell activities*. J Biol Chem.

[55] Fathali N, Ostrowski RP, Hasegawa Y, Lekic T, Tang J, Zhang JH (2013). Splenic Immune Cells in Experimental Neonatal Hypoxia–Ischemia. Transl Stroke Res.

[56] Murakami Y, Yamaguchi M, Sato T, Kobayashi R, Negishi S, Kasai K (2016). Exposure to mild hypoxia associated with Oral breathing affects the NK cell ratio in the spleen. Int J Oral-Med Sci.

[57] Mu Y, Li W, Wu B, Chen J, Chen X (2020). Transcriptome analysis reveals new insights into immune response to hypoxia challenge of large yellow croaker (Larimichthys crocea). Fish Shellfish Immunol..

[58] Ni M, Wen H, Li J, Chi M, Bu Y, Ren Y, Zhang M, Song Z, Ding H (2014). The physiological performance and immune responses of juvenile Amur sturgeon (Acipenser schrenckii) to stocking density and hypoxia stress. Fish Shellfish Immunol.

[59] Kupittayanant P, Kinchareon W (2011). Hematological and biochemical responses of the flowerhorn fish to hypoxia. J Anim Vet Adv.

[60] Goswami AR, Ghosh T (2018). Vitamin E reduces hypobaric hypoxia-induced immune responses in male rats. High Alt Med Biol.

[61] Tolonen J-P, Heikkilä M, Malinen M, Lee H-M, Palvimo JJ, Wei G-H, Myllyharju J (2020). A long hypoxia-inducible factor 3 isoform 2 is a transcription activator that regulates erythropoietin. Cell Mol Life Sci.

[62] Kristan A, Debeljak N, Kunej T (2019). Genetic variability of hypoxia-inducible factor alpha (HIFA) genes in familial erythrocytosis: analysis of the literature and genome databases. Eur J Haematol.

[63] Yamashita T, Ohneda O, Nagano M, Iemitsu M, Makino Y, Tanaka H (2008). Abnormal heart development and lung remodeling in mice lacking the hypoxia-inducible factor-related basic helix-loop-helix PAS protein NEPAS. Mol Cell Biol.

[64] Bogacheva O, Bogachev O, Menon M, Dev A, Houde E, Valoret EI, Prosser HM, Creasy CL, Pickering SJ, Grau E, Rance K, Livi GP, Karur V, Erickson-Miller CL, Wojchowski DM (2008). DYRK3 dual-specificity kinase attenuates erythropoiesis during Anemia. J Biol Chem.

[65] Geiger JN, Knudsen GT, Panek L, Pandit AK, Yoder MD, Lord KA, Creasy CL, Burns BM, Gaines P, Dillon SB, Wojchowski DM (2001). mDYRK3 kinase is expressed selectively in late erythroid progenitor cells and attenuates colony-forming unit-erythroid development. Blood..

[66] Trapnell C, Pachter L, Salzberg SL (2009). TopHat: discovering splice junctions with RNA-Seq. Bioinformatics..

[67] Li B, Dewey CN (2011). RSEM: accurate transcript quantification from RNA-Seq data with or without a reference genome. BMC Bioinformatics.

[68] Robinson MD, McCarthy DJ, Smyth GK (2010). edgeR: a bioconductor package for differential expression analysis of digital gene expression data. Bioinformatics..

[69] Kanehisa M, Goto S (2000). KEGG: Kyoto encyclopedia of genes and genomes. Nucleic Acids Res.

[70] Yu G, Wang L-G, Han Y, He Q-Y (2012). clusterProfiler: an R package for comparing biological themes among gene clusters. OMICS..

[71] Kumar L, Futschik ME (2007). Mfuzz: a software package for soft clustering of microarray data. Bioinformation..

[72] Langfelder P, Horvath S (2008). WGCNA: an R package for weighted correlation network analysis. BMC Bioinformatics..

[73] Shannon P, Markiel A, Ozier O, Baliga NS, Wang JT, Ramage D, Amin N, Schwikowski B, Ideker T (2003). Cytoscape: a software environment for integrated models of biomolecular interaction networks. Genome Res.

[74] Keenan AB, Torre D, Lachmann A, Leong AK, Wojciechowicz ML, Utti V, Jagodnik KM, Kropiwnicki E, Wang Z, Ma’ayan A (2019). ChEA3: transcription factor enrichment analysis by orthogonal omics integration. Nucleic Acids Res.

[75] Chen Z, Quan L, Huang A, Zhao Q, Yuan Y, Yuan X, et al. seq-ImmuCC: Cell-Centric View of Tissue Transcriptome Measuring Cellular Compositions of Immune Microenvironment From Mouse RNA-Seq Data. Front Immunol. 2018;9. 10.3389/fimmu.2018.01286. 10.3389/fimmu.2018.01286 PMC5996037 29922297

[76] Newman AM, Liu CL, Green MR, Gentles AJ, Feng W, Xu Y, Hoang CD, Diehn M, Alizadeh AA (2015). Robust enumeration of cell subsets from tissue expression profiles. Nat Methods.

[77] An X, Schulz VP, Li J, Wu K, Liu J, Xue F, Hu J, Mohandas N, Gallagher PG (2014). Global transcriptome analyses of human and murine terminal erythroid differentiation. Blood..

[78] Grant CE, Bailey TL, Noble WS (2011). FIMO: scanning for occurrences of a given motif. Bioinformatics..

[79] Wickham H (2011). ggplot2. WIREs Comput Stat.

[80] Chen C, Chen H, Zhang Y, Thomas HR, Frank MH, He Y, et al. TBtools: An Integrative Toolkit Developed for Interactive Analyses of Big Biological Data. Molecular Plant. 2020;13:1194–202. 10.1016/j.molp.2020.06.009 32585190

