# Supplementary material for: Exposure to hypoxia causes stress erythropoiesis and downregulates immune response genes in spleen of mice
Source: BMC Genomics. 2021 Jun 5;22:413. doi: 10.1186/s12864-021-07731-x (PMC8178839; doi:10.1186/s12864-021-07731-x)
Supplement: Supplementary file 1 — Additional file 1: Figure S1. (a) The density distribution map of transcript per million (TPM). (b) The bubble chart showing the KEGG pathways of 739 DEGs. (c) Venn diagram illustrating the overlapped KEGG pathways. (d) KEGG pathway annotation of intersection and specific parts in Figure S1c. (The color represented each comparison groups are same with Figure S1c). Figure S2. (a) Power value for the adjacency matrix in WGCNA, where the red line signals 0.85 on the vertical axis. (b) The mean connectivity of WGCNA analysis. Figure S3. (a) Hierarchical cluster tree showing coexpression modules identified by WGCNA. (b) Validation of the transcriptome data by qRT-PCR. (r were calculated by Pearson correlation). Figure S4. (a) The relative ratio of erythroid cells during terminal erythropoiesis stage. (Kruskal-Wallis test was used and P value were corrected by bonferroni) (b) Module-trait relationships plot. Each row corresponds to a module, column to different cell types during terminal erythropoiesis. (c) Heatmap of genes involved in the NF-kappa B signaling pathway. Figure S5. The correlation between erythrocyte differentiation related genes in turquoise module with Hif1a. [file 12864_2021_7731_MOESM1_ESM.pdf]

Exposure to hypoxia causes stress erythropoiesis and downregulates immune response genes in spleen of mice

Haijing Wang[1, 2, 3, 4], Daoxin Liu[1, 3, 4], Pengfei Song[1, 3, 4], Feng Jiang[1, 3, 4], Xiangwen Chi[1,3], Tongzuo Zhang [1, 3, *]

[1] Key Laboratory of Adaptation and Evolution of Plateau Biota, Northwest Institute of Plateau Biology, Chinese Academy of Sciences, Xining 810001, Qinghai, China;
[2] Medical College of Qinghai University, Xining 810016, Qinghai, China;
[3] Qinghai Provincial Key Laboratory of Animal Ecological Genomics, Xining 810008, Qinghai, China;
[4] University of Chinese Academy of Sciences, Beijing 100049, China.

*** Correspondence:**
Tongzuo Zhang
zhangtz@nwipb.cas.cn

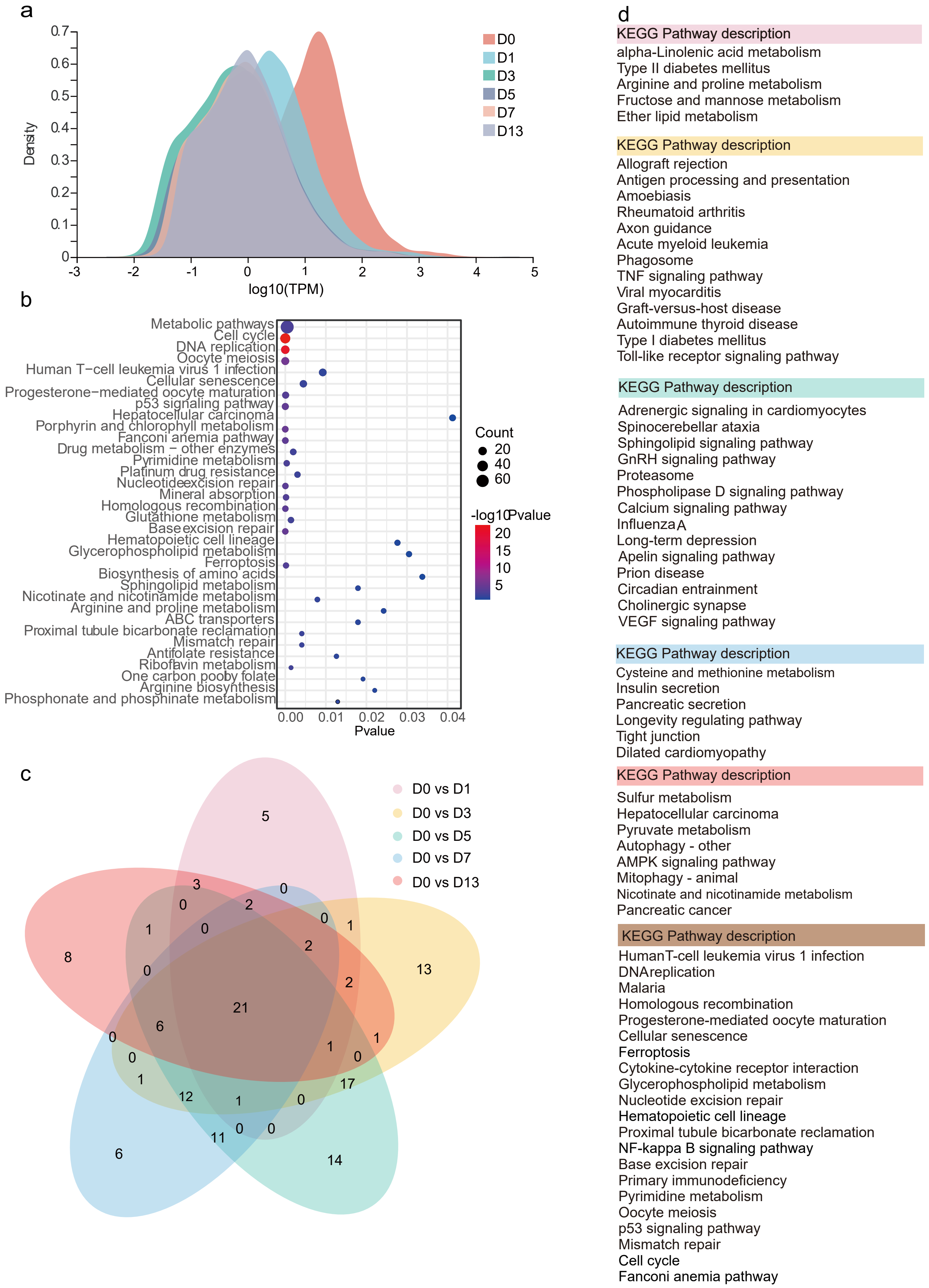

Figure S1
(a) The density distribution map of transcript per million (TPM).
(b) The bubble chart showing the KEGG pathways of 739 DEGs.
(c) Venn diagram illustrating the overlapped KEGG pathways.
(d) KEGG pathway annotation of intersection and specific parts in Figure S1c. (The color represented each comparison groups are same with Figure S1c.)

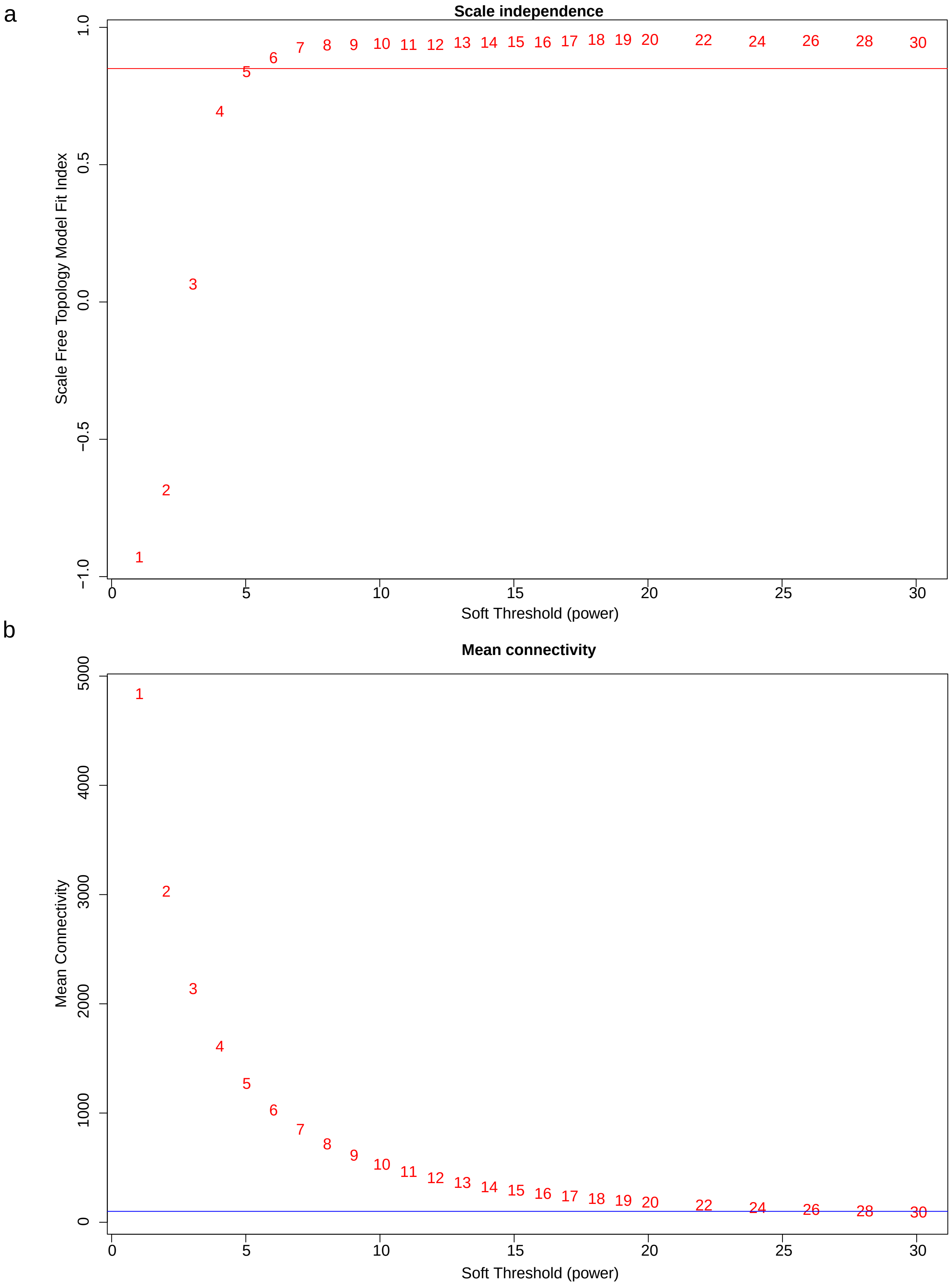

Figure S2
(a) Power value for the adjacency matrix in WGCNA, where the red line signals 0.85 on the vertical axis.
(b) The mean connectivity of WGCNA analysis.

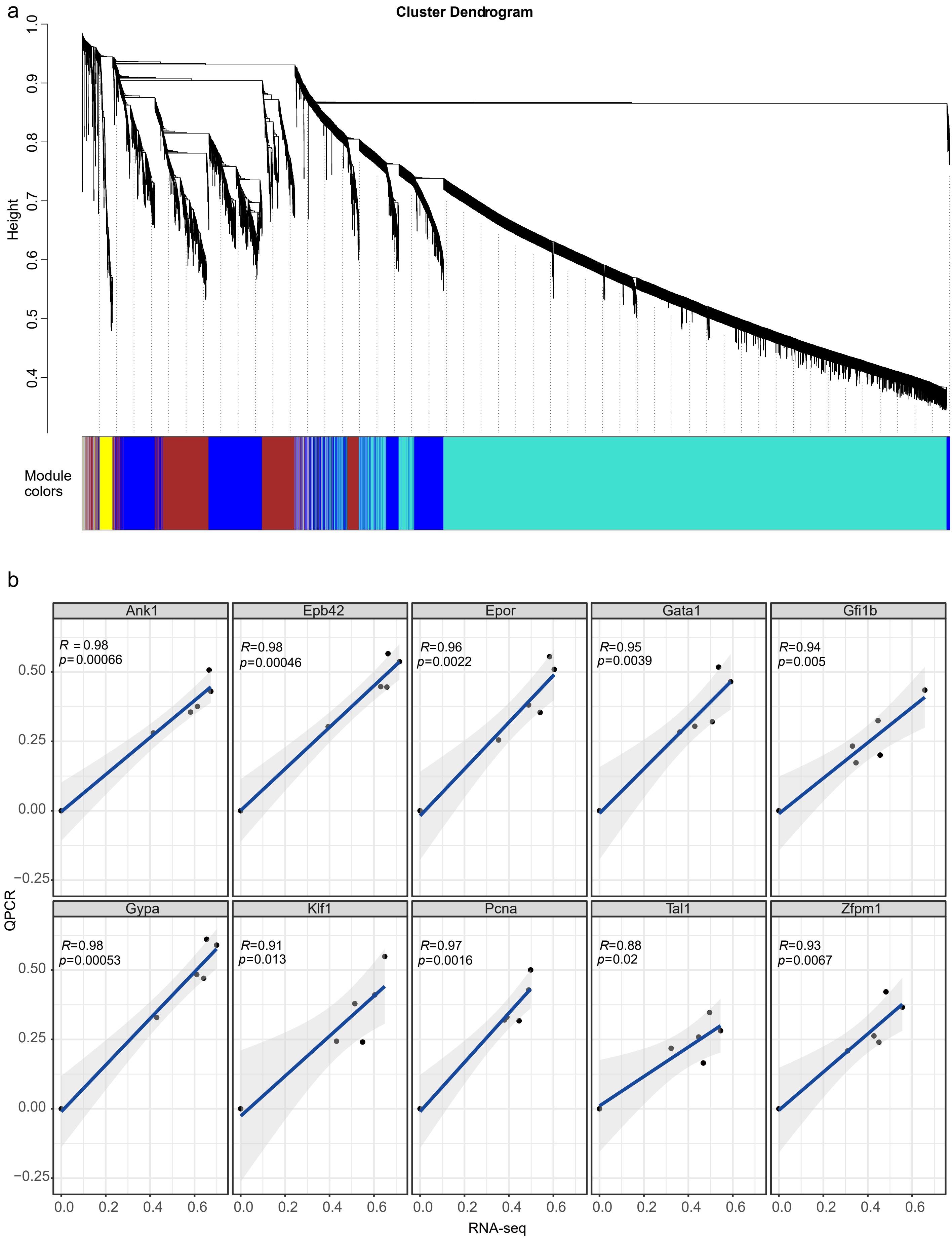

Figure S3
(a) Hierarchical cluster tree showing coexpression modules identified by WGCNA.
(b) Validation of the transcriptome data by qRT-PCR. (r were calculated by Pearson correlation).

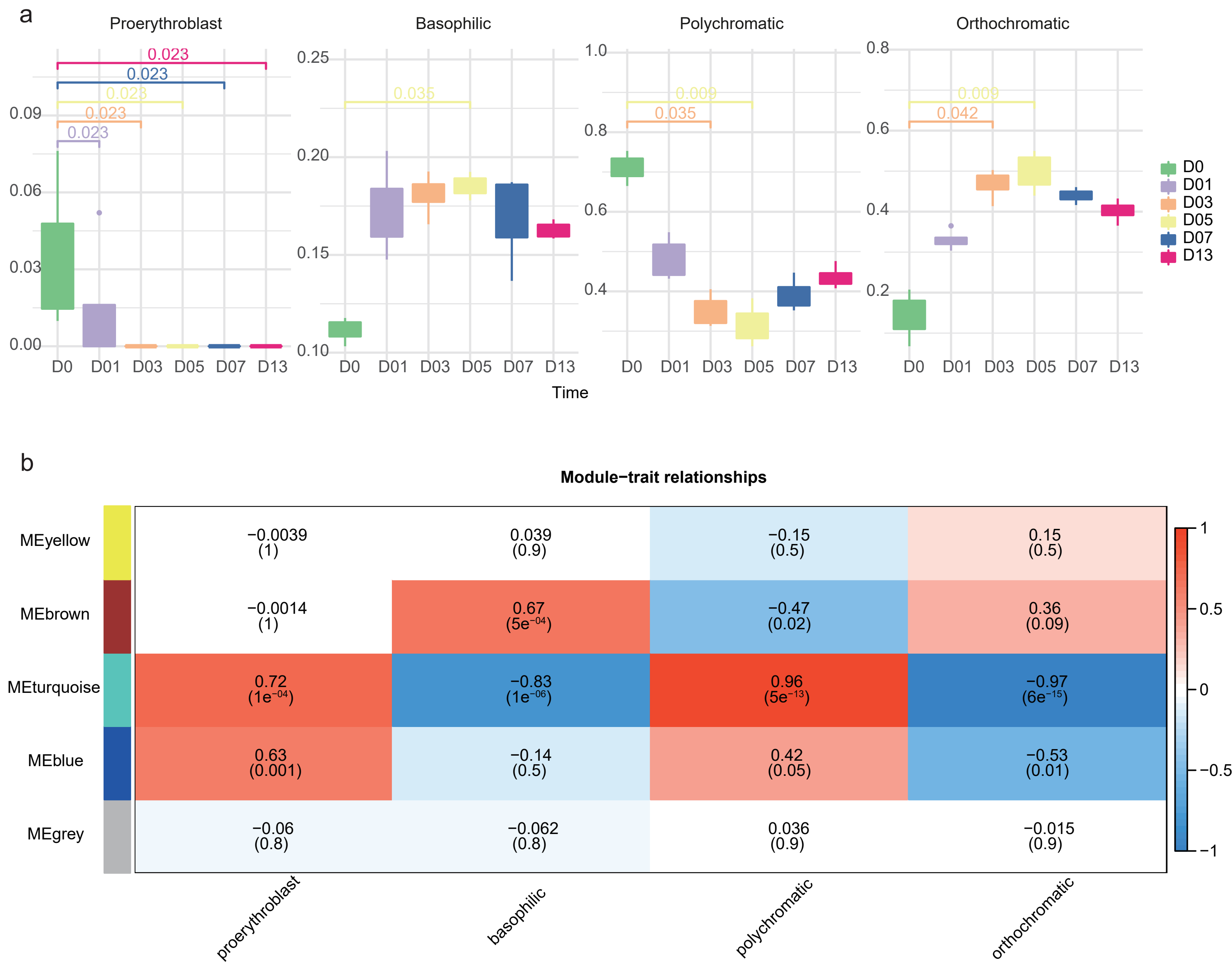

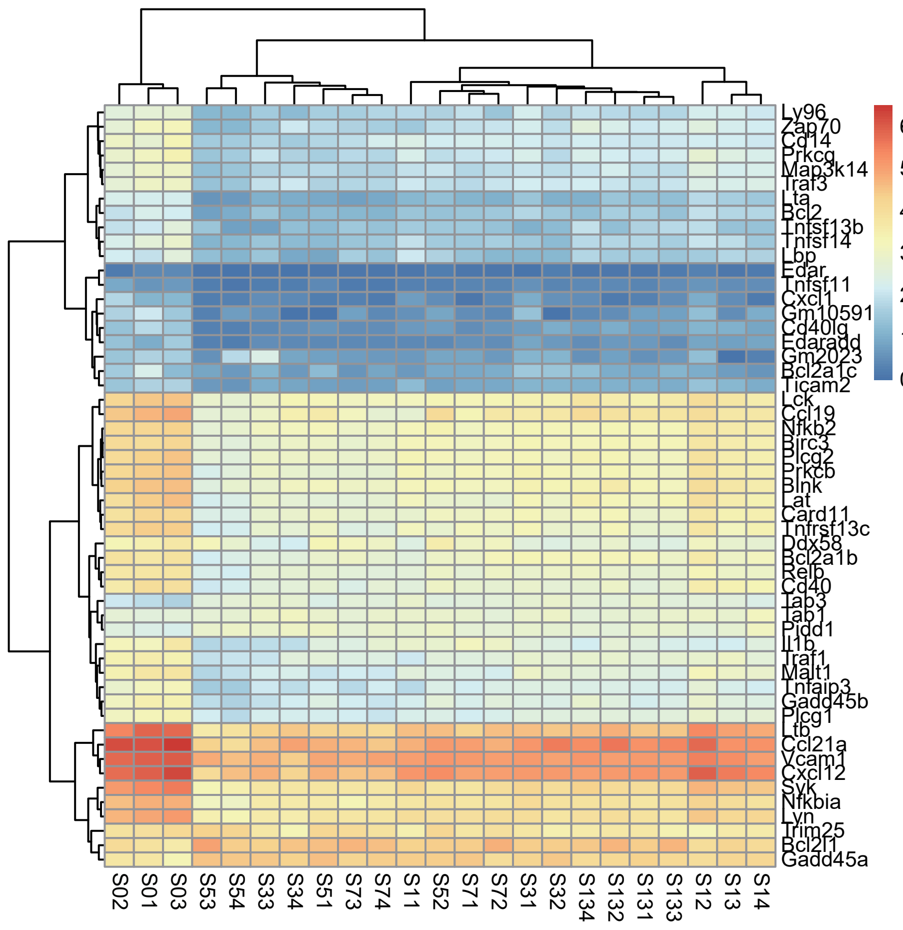

Figure S4

(a) The relative ratio of erythroid cells during terminal erythropoiesis stage. (Kruskal-Wallis test was used and *P* value were corrected by bonferroni)

(b) Module-trait relationships plot.Each row corresponds to a module, column to different cell types during terminal erythropoiesis.

(c) Heatmap of genes involved in the NF-kappa B signaling pathway.

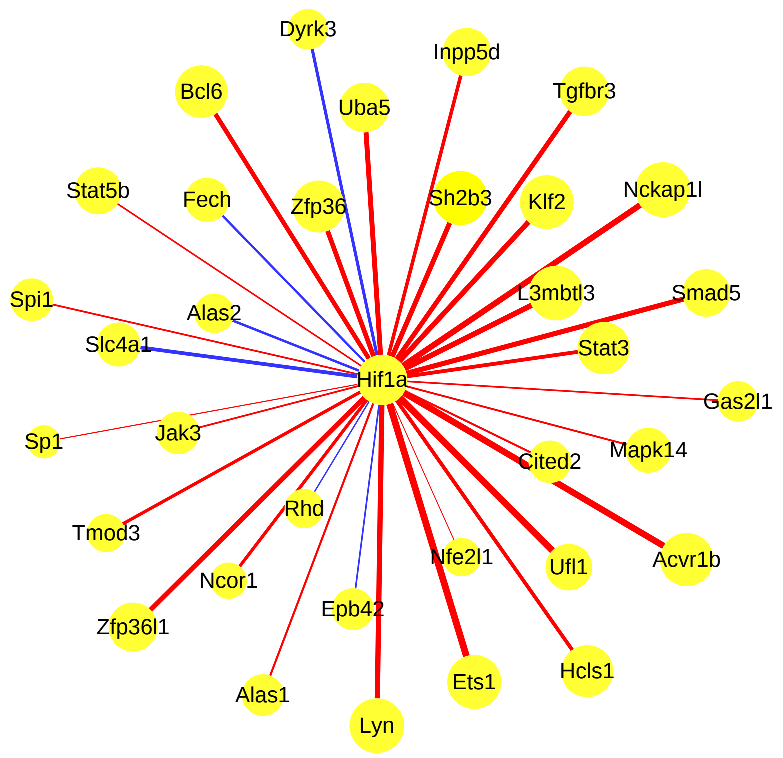

Figure S5

The correlation between erythrocyte differentiation related genes in turquoise module with Hif1a. (Blue edges indicate a negative correlation, red edges indicate a positive correlation. The width of edges indicates the correlation between nodes. The size of the node indicates its weight within turquoise module.)
